# Prevalence and Optical Coherence Tomography Biomarker Profile in Diabetic Macular Edema Among Occidental Mexican Patients

**DOI:** 10.3390/medicina62101867

**Published:** 2026-09-26

**Authors:** Diana Esperanza Arévalo-Simental, Alejandro Hernández-Rashid, Erika Martínez-López, Miriam Becerra-Cota, Gabriela Baumgarten-Robles, Liliana Gómez-Sánchez, Claudia M. Ascencio-Tene, Felipe Alexis Avalos-Salgado, Aline Priscilla Santiago-García, Carlos Barrios-González, Adolfo Daniel Rodriguez-Carrizalez

**Affiliations:** 1 Department of Ophthalmology, Hospital Civil de Guadalajara “Fray Antonio Alcalde”, Guadalajara 44280, Jalisco, Mexico; alejandrohr@live.com.mx (A.H.-R.); mgbecerra@hcg.gob.mx (M.B.-C.); gabybaumgarten@hotmail.com (G.B.-R.); dralilianagomezs@gmail.com (L.G.-S.); claudiaascenciotene@gmail.com (C.M.A.-T.); carlosbarriosgonzalez27@gmail.com (C.B.-G.); 2 Department of Physiology, Health Sciences University Center, Institute of Clinical and Experimental Therapeutics, University of Guadalajara, Guadalajara 44340, Jalisco, Mexico; adolfo.rodriguez@academicos.udg.mx; 3 Molecular Biology Department, Centro Universitario de Ciencias de la Salud, Universidad de Guadalajara, Guadalajara 44340, Jalisco, Mexico; erika.martinez@academicos.udg.mx; 4 Departamento de Farmacobiología, Centro Universitario de Ciencias Exactas e Ingenierías (CUCEI), Universidad de Guadalajara, Guadalajara 44430, Jalisco, Mexico; alexis.avalos@academicos.udg.mx; 5 Independent Researcher, Guadalajara 45030, Jalisco, Mexico; priscillasga1703@gmail.com

**Keywords:** diabetic macular edema, regional real-world study, optical coherence tomography, OCT biomarkers, intraretinal cysts, hyperreflective foci, DRIL, ellipsoid zone

## Abstract

*Background and Objectives*: Diabetic macular edema (DME) is a heterogeneous complication of diabetic retinopathy, and optical coherence tomography (OCT) biomarkers may provide information beyond central retinal thickness. Evidence on the OCT biomarker profile of Mexican patients with DME remains limited. This study aimed to characterize OCT biomarkers in patients with DME from a tertiary referral center in western Mexico and evaluate their associations with clinical characteristics and best-corrected visual acuity (BCVA). *Materials and Methods*: We conducted a retrospective cross-sectional study of adults with diabetic retinopathy and DME. Clinical records and OCT scans were reviewed. Biomarkers included central macular thickness, intraretinal cysts, subretinal fluid, hyperreflective foci, ellipsoid zone disruption, external limiting membrane disruption, and disorganization of the retinal inner layers (DRIL). Associations with glycated hemoglobin, retinopathy severity, diabetes treatment, and BCVA were analyzed. *Results*: Fifty-seven patients and 88 eyes were included. The mean age was 59.6 ± 10.3 years, the mean diabetes duration was 15.9 ± 7.1 years, and the mean HbA1c was 8.5 ± 0.9%. The mean BCVA was 1.24 ± 0.59 logMAR, and the mean central macular thickness was 427.6 ± 190.8 µm. Intraretinal cysts were the most frequent biomarker (78.4%), followed by ellipsoid zone disruption (40.9%), hyperreflective foci (34.1%), DRIL (30.7%), subretinal fluid (21.6%), and external limiting membrane disruption (21.6%). Intraretinal cysts were significantly associated with greater diabetic retinopathy severity (*p* = 0.025). *Conclusions*: This study provides a descriptive real-world profile of structural OCT biomarkers in patients with DME attending a tertiary referral center in western Mexico. Intraretinal cysts were the most frequent finding, followed by ellipsoid zone disruption, hyperreflective foci, DRIL, subretinal fluid, and external limiting membrane disruption. These findings provide regional data on the structural characteristics of DME and may serve as comparative evidence for future studies in other populations and clinical settings.

## 1. Introduction

Diabetes mellitus (DM) is a major global public health problem. According to the International Diabetes Federation, approximately 537 million people were living with diabetes worldwide in 2021 [1]. Data from the National Health and Nutrition Survey (ENSANUT) 2022 indicate that in Mexico, approximately 14.6 million individuals have diabetes [2]. The most common microvascular complication of DM is diabetic retinopathy (DR), which accounts for approximately 15% of all cases of legal blindness worldwide [3]. Diabetic macular edema (DME) is the main cause of visual disability in DM. It is estimated that approximately 20% of patients with DR will develop this condition over their lifetime [4]. From a pathophysiological perspective, DME is a multifactorial condition with a strong inflammatory component, involving numerous pro-inflammatory mediators, with vascular endothelial growth factor (VEGF) playing a central role in its pathogenesis [5] by promoting increased vascular permeability and, consequently, the development of edema due to disruption of the retinal blood barrier [6], leading to the accumulation of retinal fluid in the macula, increased retinal thickness, and vision loss [7].

The diagnosis can be clinical, and diabetic macular edema is classified by the Early Treatment Diabetic Retinopathy Study (ETDRS) as clinically significant macular edema (CSME) based on the following criteria: (1) retinal thickening within 500 μm of the center of the macula, (2) hard exudates within 500 μm of the center of the macula when associated with adjacent retinal thickening, or (3) retinal thickening involving an area greater than one disc area when located within one disc diameter of the center of the macula [8]. However, optical coherence tomography (OCT) is currently the main tool for determining the severity of DME, as it provides high-resolution cross-sectional images of the retina. Tomographic biomarkers are morphological features identified on OCT that are used to analyze prognosis, disease severity, and response to treatment [9]. Several biomarkers have been described, including central retinal thickness (CRT), intraretinal cysts, subretinal fluid, hyperreflective foci (HRF), ellipsoid zone disruption, external limiting membrane disruption, and disorganization of the retinal inner layers (DRIL), among others. Despite the growing body of evidence describing OCT biomarkers in diabetic macular edema, most studies have been conducted in Asian, European, or North American populations [9,10,11]. Consequently, the structural characteristics of diabetic macular edema in Mexican patients remain poorly characterized. Data describing the distribution of these established structural OCT biomarkers in Mexican patients also remain limited. Therefore, the aim of this study was to describe the structural OCT biomarker profile of patients with diabetic macular edema attending a tertiary referral center in western Mexico and to explore their distribution according to selected clinical characteristics.

## 2. Materials and Methods

### 2.1. Study Design and Clinical Setting

The study design was cross-sectional. We performed a systematic assessment of clinical charts from patients diagnosed with diabetic retinopathy and diabetic macular edema who attended an outpatient ophthalmology consultation from July 2023 to July 2024. The clinical records were retrospectively reviewed, and data were collected for research purposes after approval of the study protocol by the Ethics Committee in January 2025.

### 2.2. Inclusion, Exclusion and Elimination Criteria

We included patients with diabetic retinopathy and diabetic macular edema diagnosed by an ophthalmologist. Patients were eligible if they were aged ≥18 years at the time of specialist evaluation and had available optical coherence tomography (OCT) imaging data.

Patients were excluded if they had a diagnosis of primary open-angle or primary angle-closure glaucoma, neovascular glaucoma, diabetic macular edema with panretinal photocoagulation within the previous three months, or a history of phacovitrectomy performed at least six months prior to OCT acquisition. Additional exclusion criteria included OCT studies with unrecorded subsequent measurements; macular edema or retinal pathology of vascular etiology not associated with diabetes; vitreous hemorrhage preventing adequate visualization of the posterior pole or proper OCT acquisition; ocular trauma; age-related macular degeneration; retinal dystrophies; epiretinal membranes or macular holes; retinal detachment; central serous chorioretinopathy; uveitis; high myopia; anterior segment malformations; and drug-induced retinal toxicity.

### 2.3. Ethics

This study was approved by the Ethics in Research Committee (CEI) and the Committee of Research (CI) at the Hospital Civil Fray Antonio Alcalde, approval code CEI 271/24. This research protocol followed the Ethical Principles for Medical Research Involving Human Subjects described in the Helsinki Declaration [12].

### 2.4. Study Development

Demographic and clinical data were ascertained from reviewed clinical charts of patients who attended the public hospital. Information recollected was classified as follows:

Sociodemographic and comorbid disease variables: Gender, age, hypertension, type 2 diabetes mellitus (HbA1c level and duration), obesity, and other comorbid diseases.

Ophthalmologic variables: Best corrected visual acuity, lens condition, laterality, and degree of diabetic retinopathy.

Structural OCT biomarkers: Central retinal thickness, intraretinal cysts, subretinal fluid, hyperreflective foci, disruption of the ellipsoid line, disruption of the external limiting membrane, and disorganization of the inner retinal layers (DRIL). OCT images were acquired using the Cirrus HD-OCT 5000 system (Carl Zeiss Meditec, Dublin, CA, USA). All OCT scans were reviewed and interpreted by 1 ophthalmologist with subspecialty training in retina, who assessed the presence or absence of the structural OCT biomarkers included in the study. The structural OCT biomarkers were defined as follows: Central retinal thickness (CRT) was considered a quantitative parameter for monitoring retinal thickening. Intraretinal cysts were defined as hyporeflective oval spaces located within the retinal plexiform layers, whereas subretinal fluid was identified as hyporeflective spaces located beneath the hyperreflective retinal layers. Hyperreflective foci (HRF) were defined as well-demarcated hyperreflective lesions measuring approximately 20–40 µm, typically located at the level of the retinal pigment epithelium. Ellipsoid zone disruption was defined as interruption or loss of continuity of the ellipsoid zone, while external limiting membrane disruption was identified as interruption of the corresponding hyperreflective line. Disorganization of the retinal inner layers (DRIL) was defined as areas in which the normal boundaries between the inner retinal layers could not be clearly identified.

### 2.5. Statistical Analysis

The prevalence of OCT biomarkers was calculated using all eligible eyes (n = 88). To avoid the potential effect of intra-subject correlation in comparisons between clinical groups, only one eye per patient (n = 57) was included in the comparative analyses. In patients with unilateral diabetic macular edema, the affected eye was selected, whereas in patients with bilateral involvement, the right eye was preferentially selected; when the right eye was not eligible, the left eye was included. Comparisons were performed according to HbA1c levels, duration of DM2, severity of diabetic retinopathy, systemic diabetes treatment, and BCVA. Independent Student’s *t*-tests were used for comparisons of quantitative variables between groups; chi-square tests (or Fischer’s exact test if required) were used for comparisons of proportions between groups. The significance level was set at *p* ≤ 0.05. The analyses were performed using the statistical software IBM SPSS Statistics for Windows, Version 24.0 (IBM Corp., Armonk, NY, USA).

## 3. Results

A total of 57 patients with diabetic macular edema were studied. Of these, there was a greater proportion of men (56.1%) with a mean age of 59.65 ± 10.38 years. It must be noted that approximately half of the patients (49.1%) reported high blood pressure. Regarding the characteristics of diabetes, 38.5% of patients were using oral hypoglycemic drugs, 28% were receiving insulin, and 33.3% were receiving both treatments. The mean duration of the disease was 15.9 ± 7.1 years, with a mean glycated hemoglobin level of 8.5 ± 0.9% (range: 7.5–11%). Lastly, 43.8% of the subjects had proliferative diabetic retinopathy (PDR), 35.0% had inactive laser-modified PDR (LMD), and 12% had active LMD. Additionally, 21.05% had received prior laser treatment (Table 1).

Among the ophthalmological findings, 54.3% of patients had bilateral involvement, and 45.6% had unilateral involvement in the left eye (22.8%) or right eye (22.8%). The mean best-corrected visual acuity (BCVA) expressed in the LogMAR scale was 1.24 ± 0.59 for both eyes, while the mean intraocular pressure (IOP) was 14.1 ± 3.2 mmHg for both eyes. Of all eyes analyzed (n = 88), 60.2% presented with lens opacity, 10.2% had an intraocular lens, and 29% had a clear lens (Table 2).

**Table 2:** Ophthalmological characteristics.

Variable, n (%)	n = 88 Eyes (100.0)
BCVA LogMAR, mean ± SD	1.24 ± 0.59
IOP (mmHg), mean ± SD	14.1 ± 3.2
Crystalline, n (%)	
Normal	26 (29.5)
Cataract	53 (60.2)
IOL	9 (10.2)

Abbreviations: IOL—intraocular lens; IOP—intraocular pressure; mmHg—millimeters of mercury; BCVA—best corrected visual acuity; logMAR—logarithm of the minimum angle of resolution. Qualitative variables are expressed as frequencies and percentages, and quantitative variables as mean and standard deviation (SD).

Table 3 describes the characteristics of the biomarkers observed by OCT. Among the 88 eyes included in the analysis, the mean central macular thickness was 427.6 ± 190.8 µm. Intraretinal cysts were the most frequent OCT finding, present in 78.4% of cases. Interruption of the ellipsoid line was observed in 40.9% of participants, while hyperreflective foci and DRIL were identified in 34.1% and 30.7%, respectively. Subretinal fluid and interruption of the external limiting membrane were less common, each occurring in 21.6% of cases.

Presence of biomarkers were compared between different groups, such as (1) HbA1c% levels, (2) disease length, (3) severity of diabetic retinopathy, (4) diabetic treatment and (5) BCVA (logMAR) (Table 4).

## 4. Discussion

Diabetic macular edema is a heterogeneous disease, and this heterogeneity is reflected in the structural changes observed on OCT. In this study, we evaluated 57 patients and 88 eyes with DME from a tertiary referral center in western Mexico. The most frequent OCT biomarker was intraretinal cysts, followed by ellipsoid zone disruption, hyperreflective foci, DRIL, subretinal fluid, and external limiting membrane disruption. This profile should be interpreted in the context of a cohort with long-standing diabetes, suboptimal glycemic control, poor baseline visual acuity, and a high proportion of proliferative or previously laser-treated diabetic retinopathy.

Intraretinal cysts were present in 78.4% of eyes and differed significantly according to diabetic retinopathy severity. This supports the concept that cystic changes may reflect advanced blood-retinal barrier disruption rather than edema alone. Our frequency was higher than that reported by Shrivastava et al., who found intraretinal cysts with ELM disruption in 29.3% of patients [13], and also higher than the 68.2% reported by Naveen et al. in patients with DME without proliferative retinopathy [11]. However, the frequency in our cohort is consistent with the concept summarized by Sen et al., who described cystoid spaces as one of the main OCT patterns of DME, especially in eyes with chronic or more structurally advanced edema [9]. These differences may be explained by the advanced profile of our population, the high proportion of proliferative diabetic retinopathy, and methodological differences, since we recorded intraretinal cysts as present or absent regardless of their size, location, or association with ELM disruption.

Central macular thickness was increased in our cohort, with a mean value of 427.6 ± 190.8 μm. This value was comparable to previous DME cohorts, including Naveen et al., who reported a mean retinal thickness of 414.65 μm, and other OCT-based studies summarized by Sen et al., in which central macular thickness remained a key anatomical marker for diagnosis and follow-up [9,11]. However, retinal thickness alone does not fully explain visual function in DME. Sen et al. emphasized that the correlation between central macular thickness and visual acuity is often weak or inconsistent, particularly in chronic disease [9]. Similarly, Velaga et al. showed that biomarkers such as DRIL area, DRIL horizontal extent, and EZ integrity provide additional information about visual function and prognosis beyond the presence of fluid alone [14].

In agreement with this, Szeto et al. reported that baseline DRIL, retinal HRF, and ELM/EZ disruption were associated with worse visual acuity up to 24 months after anti-VEGF therapy, supporting the prognostic value of qualitative OCT biomarkers beyond retinal thickness alone [15]. Therefore, the poor BCVA observed in our study was probably multifactorial, related not only to edema but also to photoreceptor disruption, DRIL, cataract, chronicity of disease, and possible macular ischemia.

Subretinal fluid was observed in 21.6% of eyes. This proportion was higher than the 5.7% reported by Shrivastava et al. for subretinal fluid with serous retinal detachment [13], but lower than the 56.8% reported by Naveen et al. in their DME cohort [11]. These differences probably reflect variations in definition, OCT grading, and disease phenotype. In our study, subretinal fluid was considered a qualitative OCT finding, whereas other studies may restrict this category to cases with evident serous retinal detachment or may include different stages of diabetic retinopathy. Chang et al. also identified subretinal fluid as a clinically relevant biomarker associated with anatomical response after ranibizumab treatment [16]. Although our design does not allow evaluation of treatment response, the presence of subretinal fluid in approximately one fifth of eyes suggests a DME phenotype that should be explored in future longitudinal studies.

Hyperreflective foci were found in 34.1% of eyes. This frequency was higher than the 22.8% reported by Shrivastava et al. [13] but lower than the 54.4% described by Naveen et al. [11]. Comparisons between studies should be made cautiously because HRF assessment varies widely: some authors quantify the number of foci, others evaluate their location or reflectivity, and others record them qualitatively as present or absent. Tao et al. found HRF-related features to be more frequent in diabetic macular edema than in retinal vein occlusion-related macular edema [17], and Zhou et al. reported an association between HRF and systemic inflammatory markers, supporting their inflammatory relevance in DME [18].

Outer retinal biomarkers were also frequent. Ellipsoid zone disruption was present in 40.9% of eyes and ELM disruption in 21.6%. These findings suggest photoreceptor and outer retinal compromise. Saxena et al. described the ELM as a structural retinal barrier and emphasized that VEGF-related injury may lead to sequential ELM and EZ disruption [19]. Sen et al. also highlighted photoreceptor integrity as a relevant biomarker for visual prognosis in DME [9]. In addition, Velaga et al. reported that baseline BCVA worsened progressively with decreasing EZ integrity, supporting the functional relevance of outer retinal damage [14]. Viggiano et al. also found that, in eyes with resolved DME after anti-VEGF therapy, ELM status remained associated with visual acuity, suggesting that outer retinal integrity may continue to influence visual function even after edema resolution [20]. In our cohort, EZ and ELM abnormalities were frequent in this cohort, although their distribution did not differ significantly according to BCVA categories.

DRIL was present in 30.7% of eyes, similar to the 31% reported by Shrivastava et al. for DRIL with ELM disruption [13], and close to the 38.6% reported by Naveen et al. [11]. DRIL is considered a marker of inner retinal disorganization and visual dysfunction [21]. Chang et al. reported that DRIL may predict anatomical response after ranibizumab treatment [16], while Velaga et al. found that DRIL area and maximum horizontal extent were associated with baseline BCVA and visual prognosis after treatment [14]. Viggiano et al. also identified DRIL as one of the qualitative OCT features most strongly associated with BCVA in eyes with resolved DME after anti-VEGF therapy [20]. More recently, Tripathi et al. summarized evidence supporting DRIL as a marker of inner retinal structural damage, visual prognosis, and possibly macular ischemia in diabetic retinal disease [22]. In our cohort, DRIL was numerically more frequent in eyes with worse BCVA, although the association was not statistically significant.

Diabetes duration was not significantly associated with the presence of any of the evaluated OCT biomarkers. Although some biomarkers showed numerical differences between patients with a disease duration of ≤10 years and those with ≥11 years, none of these differences reached statistical significance. Similarly, the type of systemic treatment for diabetes, including oral hypoglycemic agents, insulin, or a combination of both, was not significantly associated with the distribution of OCT biomarkers. These findings suggest that, in this cohort, no significant differences in OCT biomarker distribution were observed according to diabetes duration or systemic diabetes treatment in this cohort.

A strength of this study is the availability of real-world data from a Mexican tertiary referral center, providing regional information from a clinical setting that has been less frequently represented in the literature on OCT biomarkers in DME. The simultaneous description of several established structural OCT features provides a comprehensive profile of the retinal findings observed in this cohort. In addition, the study assessed several clinically relevant biomarkers, including fluid-related, inflammatory, outer retinal, and inner retinal structural markers. This allows a broader characterization of DME than central macular thickness alone. Another strength is that all patients were evaluated in a referral setting with a high burden of advanced diabetic retinopathy, which reflects the type of patients frequently encountered in public ophthalmology services.

In our cohort, central macular thickness was useful to confirm edema, but it did not capture the full spectrum of retinal damage seen on OCT. Intraretinal cysts and subretinal fluid reflected barrier dysfunction and fluid accumulation; HRF may reflect inflammatory or lipid-related activity; EZ and ELM disruption suggested outer retinal damage; and DRIL reflected inner retinal disorganization. This structural phenotyping may be especially useful in referral settings where patients often present with advanced diabetic retinopathy. Characterizing this structural profile in a regional Mexican tertiary-care cohort provides descriptive data that may be useful for comparison with DME populations evaluated in other clinical and geographic settings.

This study has limitations. Its cross-sectional design prevents evaluation of causality, progression, or treatment response. The sample size was modest, although the most relevant systemic variables were available for the majority of patients. Optical coherence tomography angiography (OCTA) or fluorescein angiography was not performed because the primary objective was to assess cross-sectional neuroretinal reflectivity and architecture, rather than capillary perfusion or macular ischemia. Furthermore, the presence of severe macular edema can induce significant signal attenuation, shadowing, and projection artifacts within cystoid spaces, which frequently compromises the segmentation accuracy and quantitative reliability of OCTA plexuses. Another limitation of this study is that the OCT biomarkers evaluated are not novel and have already been described in other populations. In addition, the study was conducted in a single Mexican tertiary referral center, which may limit the generalizability of the findings to other populations and healthcare settings. Nevertheless, the characterization of these biomarkers in an underrepresented population adds complementary evidence to the existing literature and may contribute to a broader understanding of the structural heterogeneity of diabetic macular edema across different clinical and demographic contexts.

This study focused exclusively on the characterization of structural OCT biomarkers—such as intraretinal fluid (IRF), subretinal fluid (SRF), disorganization of the retinal inner layers (DRIL), and hyperreflective foci (HRF)—to evaluate macular morphology in Mexican patients with diabetic macular edema. Therefore, morphological evaluation via structural B-scans was prioritized to ensure data homogeneity and reproducible biomarker analysis.

## 5. Conclusions

In this study, we characterized key structural OCT biomarkers in diabetic macular edema, including intraretinal cysts, subretinal fluid, hyperreflective foci, ellipsoid zone disruption, external limiting membrane disruption, and DRIL. The most prevalent structural biomarkers in this cohort of patients with long-standing diabetes mellitus were intraretinal cysts, ellipsoid zone disruption, and hyperreflective foci. Intraretinal cysts differed significantly according to diabetic retinopathy severity, suggesting a relationship between this structural finding and the ophthalmologic stage of the disease in this study. These findings provide a descriptive regional profile of structural OCT characteristics in patients with DME from a tertiary referral center in western Mexico. Further longitudinal studies are warranted to confirm these observations and explore their relationship with disease progression and treatment response.

## Figures and Tables

**Table 1:** Sociodemographic characteristics of patients with diabetic macular edema.

Variable, n (%)	n = 57 (100.0)
Gender (%)	
Male	32 (56.1)
Female	25 (43.8)
Age (years), mean ± SD	59.6 ± 10.3
Systemic arterial hypertension, n (%)	28 (49.12%)
Treatment for diabetes, n (%)	
Oral hypoglycemic drugs	22 (38.5)
Insulin	16 (28.2)
Both	19 (33.3)
Diabetes mellitus length (years), mean ± SD	15.9 ± 7.1
HbA1c% levels, mean ± SD	8.5 ± 0.9
Severity of diabetic retinopathy, n (%)	
Mild	1 (1.7)
Moderate	1 (1.7)
Severe	3 (5.2)
PDR	25 (43.8)
Previously laser-treated diabetic retinopathy with activity	7 (12.2)
Previously laser-treated diabetic retinopathy without activity	20 (35.0)
Previous laser treatment, n (%)	12 (21.0)

Qualitative variables are expressed as frequencies and percentages, and quantitative variables as means and standard deviations (SDs). Abbreviations: PDR, proliferative diabetic retinopathy; HbA1c, glycated hemoglobin or hemoglobin A1c.

**Table 3:** Macular OCT biomarker descriptions.

Variable, n (%)	n = 88 (100.0)
Central macular thickness, mean ± SD	427.6 ± 190.8
Intraretinal cysts, n (%)	69 (78.4)
Subretinal fluid, n (%)	19 (21.6)
Hyperreflective foci, n (%)	30 (34.1)
Interruption of ellipsoid line, n (%)	36 (40.9)
Interruption of external limiting membrane, n (%)	19 (21.6)
DRIL, n (%)	27 (30.7)

Abbreviation: DRIL—disorganization of the inner retina layers. Qualitative variables are expressed as frequencies and percentages, and quantitative variables as mean and standard deviation (SD).

**Table 4:** Comparison of OCT biomarkers in patients with diabetic macular edema.

Variable, n (%)	n	Intraretinal Cysts	Subretinal Fluid	Hyperreflective Foci	Interruption of Ellipsoid Line	Interruption of External Limiting Membrane	DRIL
HbA1c% *		15 (100.0)	2 (100.0)	7 (100.0)	8 (100.0)	6 (100.0)	10 (100.0)
7.5 to 8.4%	8	7 (46.7)	1 (50.0)	3 (42.9)	3 (37.5)	3 (50.0)	6 (60.0)
8.5 to 11.0%	9	8 (53.3)	1 (50.0)	4 (57.1)	5 (62.5)	3 (50.0)	4 (40.0)
*p*-value		0.9	0.9	0.7	0.4	0.8	0.2
Disease length		48 (100.0)	14 (100.0)	24 (100.0)	28 (100.0)	17 (100.0)	21 (100.0)
≤10 years	14	11 (22.9)	3 (21.4)	8 (33.3)	4 (14.3)	4 (23.5)	6 (28.6)
≥11 years	43	37 (77.1)	11 (78.6)	16 (66.7)	24 (85.7)	13 (76.5)	15 (71.4)
*p*-value		0.5	0.7	0.1	0.07	0.9	0.5
Severity		48 (100.0)	14 (100.0)	24 (100.0)	28 (100.0)	17 (100.0)	21 (100.0)
NPDR	5	2 (4.2)	1 (7.1)	3 (12.5)	3 (10.7)	1 (5.9)	2 (9.5)
PDR	24	22 (45.8)	7 (50.0)	9 (37.5)	13 (46.4)	7 (41.2)	8 (38.1)
PDR with previous PRP, active	8	6 (12.5)	4 (28.6)	4 (16.7)	4 (14.3)	2 (11.8)	3 (14.3)
PDR with previous PRP inactive	20	18 (37.5)	2 (14.3)	8 (33.3)	8 (28.6)	7 (41.2)	8 (38.1)
*p*-value		**0.025**	0.1	0.7	0.7	0.9	0.9
Diabetes treatment		48 (100.0)	14 (100.0)	24 (100.0)	28 (100.0)	17 (100.0)	21 (100.0)
Oral hypoglycemic agent	22	16 (33.3)	6 (42.9)	10 (41.7)	8 (28.6)	5 (29.4)	8 (38.1)
Insulin	16	15 (31.3)	3 (21.4)	8 (33.3)	9 (32.1)	6 (35.3)	7 (33.3)
Hypoglycemic agent + insulin	19	17 (35.4)	5 (35.7)	6 (25.0)	11 (39.3)	6 (35.3)	6 (28.6)
*p*-value		0.1	0.8	0.5	0.3	0.6	0.7
BCVA (logMAR)		48 (100.0)	14 (100.0)	24 (100.0)	28 (100.0)	17 (100.0)	21 (100.0)
0.3 to 1.0	34	27 (53.3)	8 (57.1)	17 (70.8)	16 (57.1)	10 (58.8)	12 (57.1)
1.1 to 2.6	23	21 (43.8)	6 (42.9)	7 (29.2)	12 (42.9)	7 (41.2)	9 (42.9)
*p*-value		0.2	0.8	0.1	0.7	0.9	0.7

Qualitative variables are expressed as frequencies. Statistical differences were calculated using the Chi square test. Abbreviations: NPDR, non-proliferative diabetic retinopathy; PDR, proliferative diabetic retinopathy; PRP, panretinal photocoagulation; BCVA, best corrected visual acuity, HbA1c, glycated hemoglobin or hemoglobin A1c. * HbA1c% data were available for 17 patients.

## Data Availability

The database supporting the findings of this study is available upon reasonable request by contacting the corresponding author.
